# Ubiquitin-like prokaryotic MoaD as a fusion tag for expression of heterologous proteins in *Escherichia coli*

**DOI:** 10.1186/1472-6750-14-5

**Published:** 2014-01-21

**Authors:** Sujuan Yuan, Xin Wang, Jian Xu, Zheng Yan, Nan Wang

**Affiliations:** 1 Chinese Academy of Medical Sciences & Peking Union Medical College, Institute of Materia Medica, Beijing Key Laboratory of New Drug Mechanisms and Pharmacological Evaluation Study, Beijing, People’s Republic of China

**Keywords:** MoaD, Prokaryote, Ubiquitin, Expression, Degradation, Protein folding

## Abstract

**Background:**

Eukaryotic ubiquitin and SUMO are frequently used as tags to enhance the fusion protein expression in microbial host. They increase the solubility and stability, and protect the peptides from proteolytic degradation due to their stable and highly conserved structures. Few of prokaryotic ubiquitin-like proteins was used as fusion tags except ThiS, which enhances the fusion expression, however, reduces the solubility and stability of the expressed peptides in *E. coli*. Hence, we investigated if MoaD, a conserved small sulfur carrier in prokaryotes with the similar structure of ubiquitin, could also be used as fusion tag in heterologous expression in *E. coli*.

**Results:**

Fusion of MoaD to either end of EGFP enhanced the expression yield of EGFP with a similar efficacy of ThiS. However, the major parts of the fusion proteins were expressed in the aggregated form, which was associated with the retarded folding of EGFP, similar to ThiS fusions. Fusion of MoaD to insulin chain A or B did not boost their expression as efficiently as ThiS tag did, probably due to a less efficient aggregation of products. Interestingly, fusion of MoaD to the murine ribonuclease inhibitor enhanced protein expression by completely protecting the protein from intracellular degradation in contrast to ThiS fusion, which enhanced degradation of this unstable protein when expressed in *E. coli*.

**Conclusions:**

Prokaryotic ubiquitin-like protein MoaD can act as a fusion tag to promote the fusion expression with varying mechanisms, which enriches the arsenal of fusion tags in the category of insoluble expression.

## Background

Fusion expression is a common strategy in the production of recombinant proteins. Various fusion tags are used to enhance the total and soluble expression yield in *E. coli*. Fusion tags show differing efficiency in enhancing the expression, solubility and stability of recombinant proteins [1-5]. The stable or conserved structure of the fusion tag is speculated as a determinant of its fusion properties [6].

Ubiquitin (Ub) and SUMO are stable, highly conserved small proteins expressed in all eukaryotic cells. They are frequently used as tags to enhance the fusion expression by increasing the solubility and stability of the expressed peptides and protecting the peptides from proteolytic degradation in prokaryotic host [6-8]. MoaD and ThiS, components of prokaryotic sulfur transfer systems, are also highly conserved small proteins found in prokaryotic cells. They display a high degree of structural similarity although sharing limited sequence similarities to Ub, and interact with correlating enzymes in similar ways as Ub [9-12]. They are Ub-like proteins (Ubl) and have been suggested as prokaryotic antecedents of Ub. Prokaryotic ThiS was also tried as a fusion tag in expression of heterologous proteins in *E. coli*[13]. It was able to induce aggregation of fusion proteins due to a slowdown of refolding, and enhance the expression of some targets more significantly than its eukaryotic counterpart Ub. But it promoted the degradation of unstable target fusion.

In this report, we observed the effect of fusion of MoaD, a small prokaryotic ubl containing 81 amino acid residues, on the expression of several targets in *E. coli*. MoaD showed the similar properties to prokaryotic ubl ThiS in enhancing the recombinant expression and promoting the aggregation of fusion proteins through slowdown of target folding. Contrary to ThiS fusion, MoaD fusion conferred a complete protection of the murine ribonuclease inhibitor (mRI) from intracellular degradation.

## Results

### MoaD fusion enhances the expression of EGFP

**Figure 1:**
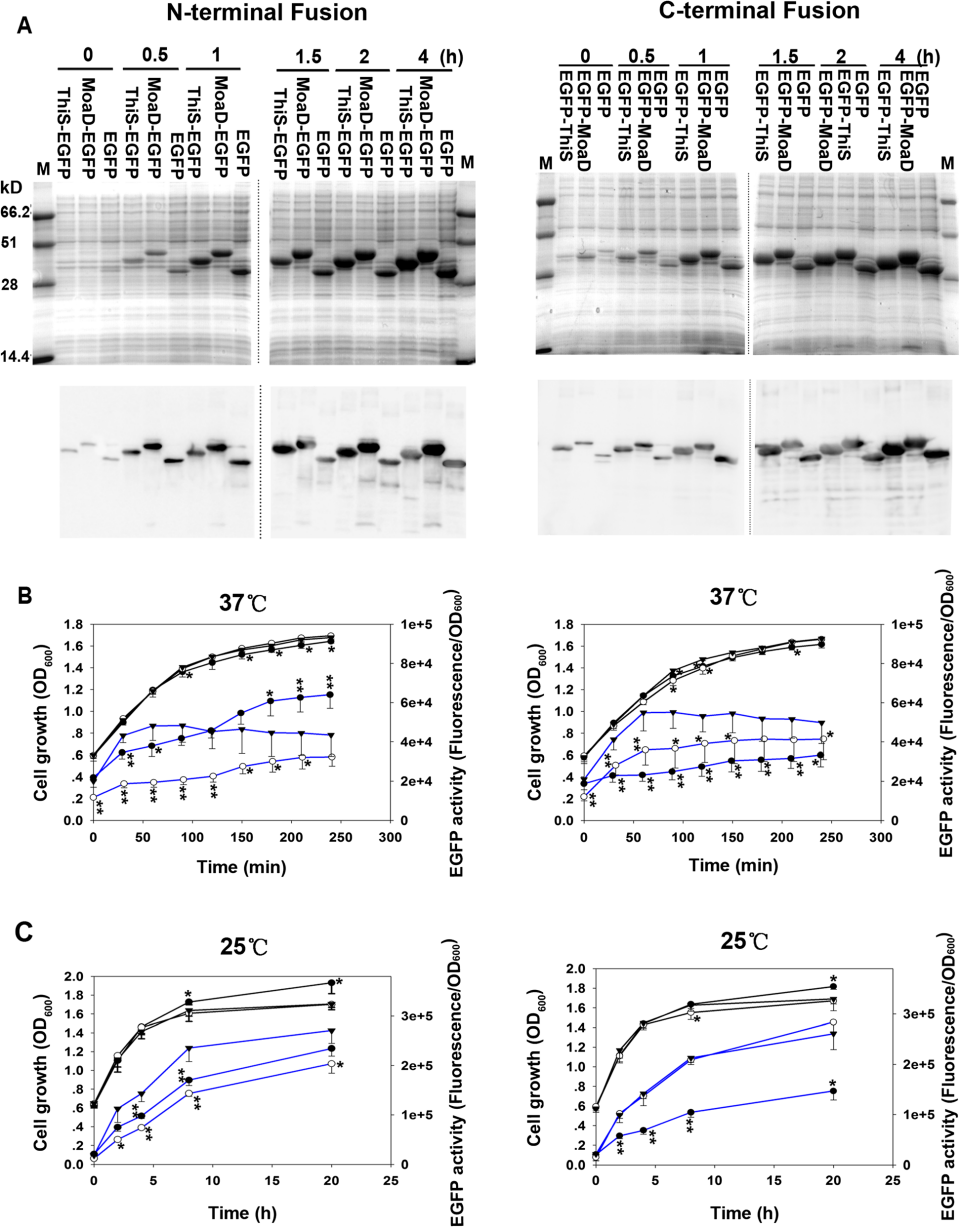
**Enhanced expression of EGFP fused with MoaD. (A)** The recombinant EGFP proteins without fusion (EGFP), and with MoaD-tag fused at N-terminus (left panels) or C-terminus (right panels), were induced by 1 mM IPTG for 4 h at 37°C. ThiS-tag fusions were used as positive control. Total cell lysates were resolved on 12% SDS-PAGE (upper panels) and analyzed by immunoblot with anti His-tag antibody (lower panels). **(B)** At 37°C induced by 1 mM IPTG or **(C)** at room temperature induced by 0.1 mM IPTG, cell growth (black lines) was recorded by measuring absorbance at 600 nm, and the fluorescence of cells was measured (excitation 488 nm; emission 509 nm) and normalized to corresponding OD_600_ (blue lines). Triangle is for EGFP, open circle for ThiS fusion, and solid circle for MoaD fusion. Each point represents mean and SD of 4 independent experiments. *p < 0.05; **p < 0.01 comparing to EGFP control.

The gene encoding EGFP was fused in frame to the gene of MoaD either at upstream or at downstream, and was cloned into prokaryotic expression vector pQE30. Similar constructs via ThiS fusion were used as the controls. All these MoaD-fused EGFP were expressed more abundantly than EGFP alone in *E. coli* TG1 at 37°C (Figure 1A).

The fluorescence of cells expressing EGFP with or without fusions was measured after IPTG induction. The intensity of fluorescence increased steadily at 37°C. The fluorescence was normalized to the measured OD_600_, since a slight but significant difference in OD_600_ was noticed among the cells, although they grew at the similar rate (Figure 1B). The normalized intensity of fluorescence thus was in direct proportion to the emitted fluorescence of single cell in average. The fluorescence of the cells bearing EGFP alone reached to a plateau after 1 h induction at 37°C (Figure 1B). N-terminal MoaD fusion gave a continued increase in normalized fluorescence which was much higher than EGFP alone after 2 h induction at 37°C. N-terminal ThiS fusion emitted much lower fluorescence. The cells bearing C-terminal fusion of MoaD had significantly lower fluorescence than cells bearing EGFP alone. C-terminal fusion of ThiS also caused significantly lower fluorescence than EGFP alone, consisting to the previous report [13].

Since the accumulation of non-native EGFPs in inclusion bodies could have reduced the measured fluorescence, we did similar experiments with lower concentration of inducer and under lower cultured temperature in expecting to improve the soluble expression and reduce the inclusion body formation. Indeed, higher fluorescence was reached for all the recombinants (Figure 1C). Nevertheless, much less fluorescence had been observed in both N-terminal MoaD fusion and N-terminal ThiS fusion than EGFP alone. Interestingly, even less fluorescence was observed in the C-terminal MoaD fusion under the same conditions. Whereas the C-terminal ThiS fusion produced an identical fluorescence as did EGFP alone at room temperature. The discrepancy in growing fluorescence may reflect the difference in relative amount of soluble active proteins and insoluble fluorescent folding intermediates, as previously identified [13].

### MoaD fusion promotes the aggregation of EGFP

**Figure 2:**
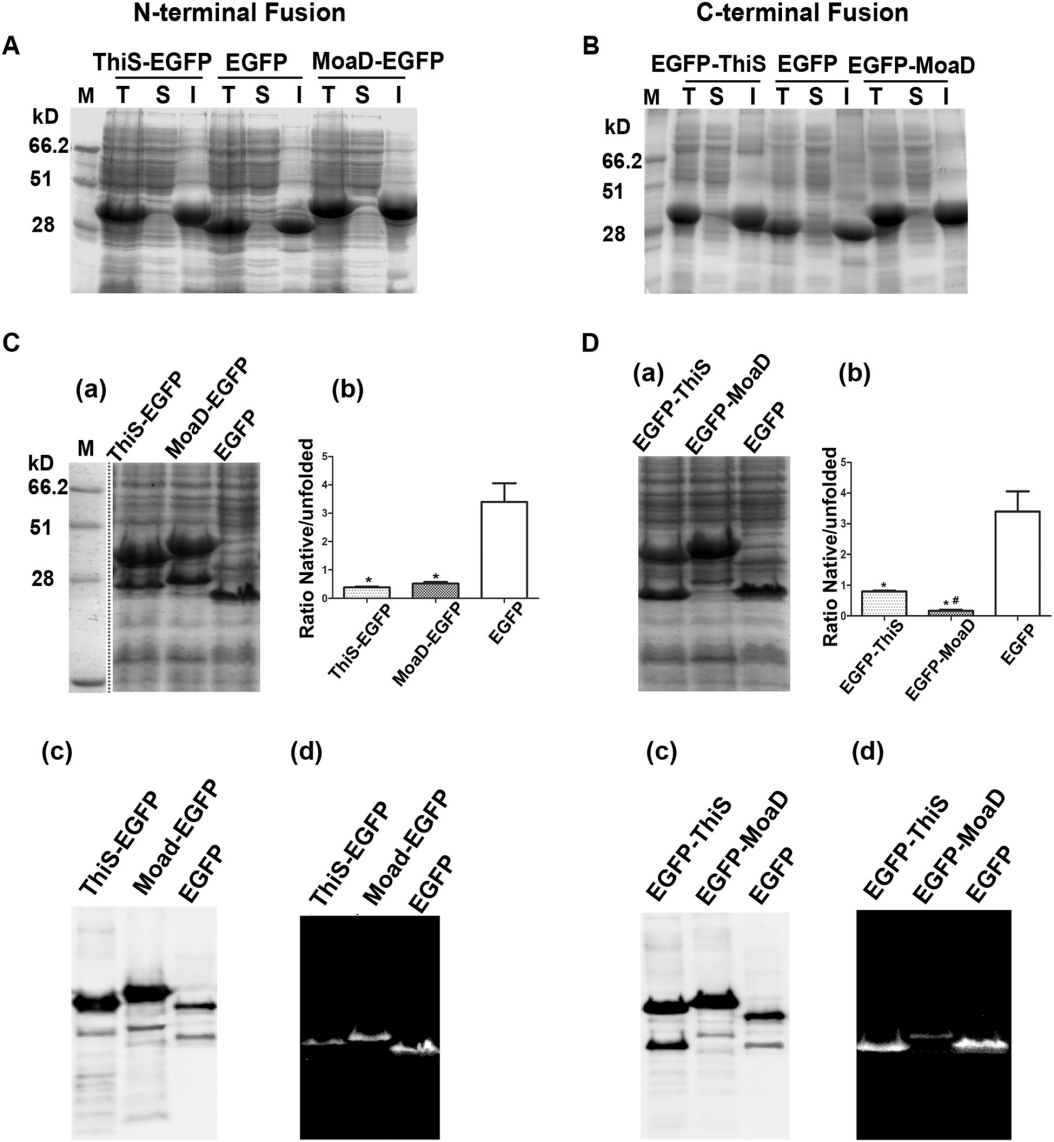
**Enhanced aggregation of EGFP in fusion with MoaD.** In the upper panel, the recombinant proteins of EGFP fused with MoaD at N-terminus **(A)** or C-terminus **(B)**, were induced by 1 mM IPTG for 4 h at 37°C. EGFP alone without fusion, and fusions with ThiS were used as control. Total cell lysate (T) and the soluble (S) or insoluble (I) fractions were resolved on 12% SDS-PAGE. All the proteins were mainly expressed in the inclusion bodies. In the lower panel, the cells were induced with 0.1 mM IPTG at room temperature for 20 h. Unboiled total cell lysates of fusions at N-terminus **(C)** or C-terminus **(D)** were resolved on 12% SDS-PAGE. The soluble native form was separated from insoluble denatured form for each sample **(a)**. Their ratios were calculated and compared to that of EGFP **(b)**, *p < 0.05 for triplicate experiments; # *p* < 0.05 for comparison between ThiS- and MoaD-fusion. The Western blot with His-tag antibody **(c)** and UV illuminated gel **(d)** further confirmed the identities of overexpressed products and corresponding native forms.

To verify whether the difference in fluorescence growing patterns could be attributed to the differential soluble expression of the fusions, cells were analyzed for their expression in native form in soluble portion and non-native one in aggregation by SDS-PAGE. We found that EGFPs with or without fusions were expressed in inclusion bodies at 37°C (Figure 2A and B). Similarly, we analysed the whole cell lysates from milder induction at lower cultured temperature by SDS-PAGE without boiling denaturation, which separated the native EGFP proteins in soluble form from their non-native molecules in inclusion bodies [13]. The EGFP without tag was predominantly expressed in native form at room temperature (Figure 2C and D). Major parts of N-terminal and C-terminal fused EGFP with MoaD were expressed in non-native form as compared to EGFP without tag. C-terminal fusion of MoaD had even much less native active fluorescent protein compared to that of EGFP alone and ThiS fusion, which was consistent to the observation that much less *in vivo* fluorescence of C-terminal MoaD fusion was induced at room temperature (Figure 1C).

**Figure 3:**
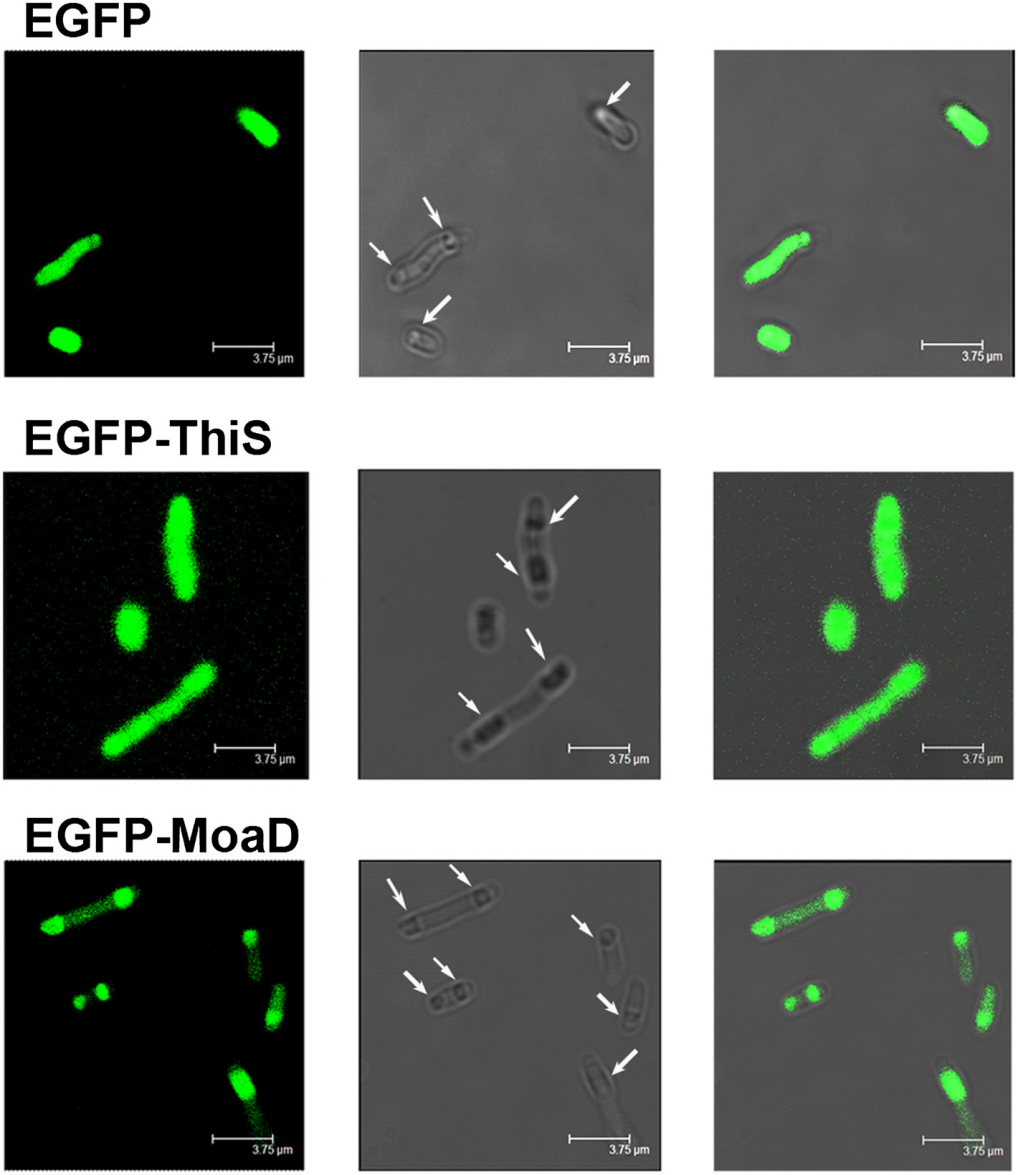
**Fluorescence distribution of EGFP fusion proteins within *E. coli.****E. coli* TG1 bearing EGFP without fusion or with C-terminal ThiS or MoaD fusion was cultured overnight at 37°C without IPTG induction. Cells on cover slide were subjected to confocal microscopy with laser excitation at 488 nm. Representative photos were shown in left panels as fluorescence images, middle panels as phase contrast images with inclusion bodies indicated by arrows, and right panels as their merged images. Scale bar in each photo represents 3.75 μm.

To further elucidate the difference in soluble expression of C-terminal fusion of MoaD from that of ThiS fusion and EGFP alone, we analysed the fluorescence distribution within cells by confocal microscopy. As shown in Figure 3, the inclusion bodies were present within all the cells even under leaky expression conditions. The fluorescence was evenly distributed in the cells expressing EGFP alone or with ThiS fusion. The fluorescence in cells with C-terminal MoaD fusion was chiefly segregated in the inclusion bodies (Figure 3 lowest panel). This was consistent with the observation illustrated in Figure 2D.

These results suggest that fusion of EGFP with MoaD at either N- or C-terminus enhances the expression of the fusion protein which presents mostly as aggregated inclusion bodies in a similar way as fusions with ThiS.

### MoaD fusion retards the refolding of EGFP

**Figure 4:**
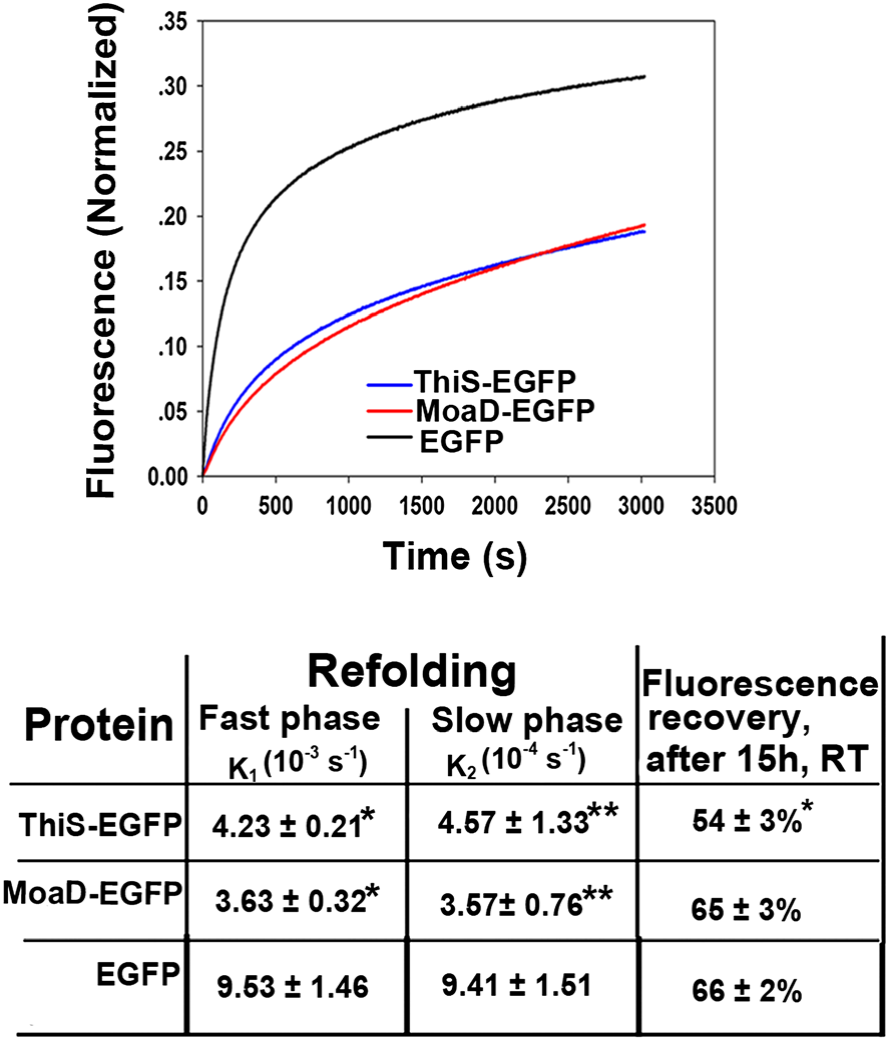
***In vitro *refolding of EGFP in fusion with MoaD.** The refolding kinetics of N-terminal fusion of EGFP with MoaD (red line) and ThiS (blue line) were compared *in vitro* to that of EGFP without fusion (black line) in the upper panel, which represents an averaged result of the short term refolding curves from three independent experiments, with fluorescence (normalized to the respective final fluorescence recovered) plotted against time. In the lower panel, kinetics of an initial fast refolding phase, the following slow refolding phase, and the percentage of refolding at final stage (15 h) were compared to EGFP control. *p < 0.05; **p < 0.01

The enhanced aggregation of EGFP by C-terminal ThiS fusion was previously attributed to the reduced refolding rate of EGFP [13]. Hence, we evaluated the foldability of N-terminal fusion of EGFP with MoaD or ThiS. Purified fluorescent EGFP with fusions was denatured and renatured *in vitro*. Upon dilution, both fused proteins refolded gradually with an increase in fluorescence, the same as EGFP alone (Figure 4). MoaD-fused EGFP had the same final recovery of fluorescence as did EGFP alone, whereas ThiS-fused EGFP had a slightly lower final recovery of fluorescence. Both MoaD- and ThiS-fused EGFP refolded at a significantly slower rate either in fast or slow refolding phases (indicated by lower k_1_ and k_2_ values in Figure 4). The soluble fluorescent C-terminal MoaD fused EGFP was subjected to rapid *in vitro* fragmentation after purification (Additional file 1: Figure S1), hence its foldability was not identified.

### MoaD is inefficient in enhancing the expression of insulin A and B chains

**Figure 5:**
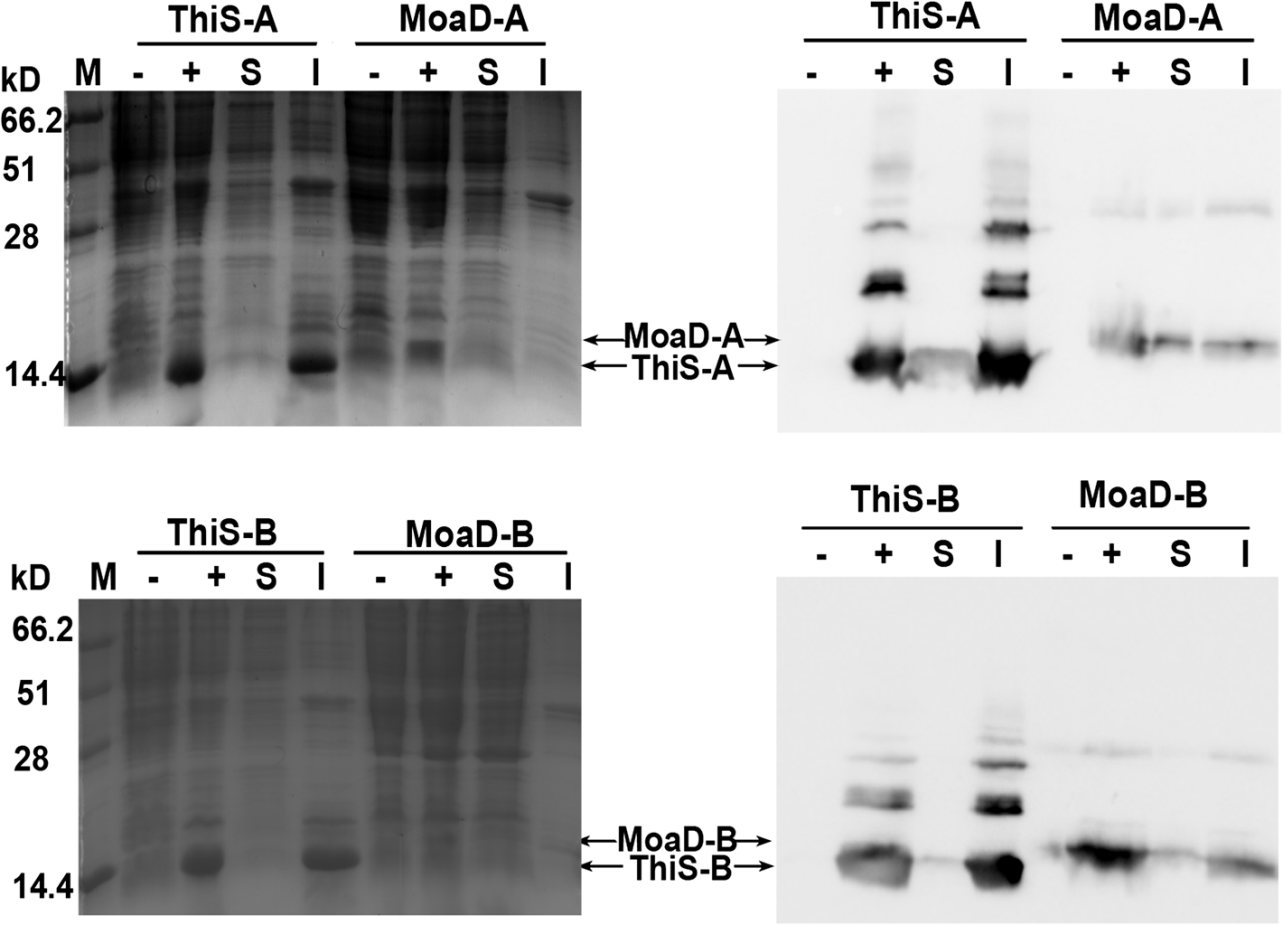
**Expression of insulin chains with MoaD fusion.** Insulin A chain (upper panel) or B chain (lower panel) fused with MoaD at their N-termini, were expressed in *E. coli* BL21 (DE3) pLysS. ThiS-fused chains were used as control. Total cell lysate from uninduced (−) or induced (+) cells with IPTG, and the soluble (S) or insoluble fraction (I) of induced cells were electrophoresed on 15% SDS-PAGE, shown in left panel. Expressed proteins were verified by Western blot probed with anti His-tag antibody, shown in right panel. Arrows highlight expressed proteins at expected positions.

Since ThiS fusion enhanced the expression of insulin chain A and B [13], we also fused the gene of MoaD to the gene encoding insulin chain A or B at their upstream and cloned into prokaryotic expression vector pET28a. The fusion proteins were expressed in much less amount in *E. coli* BL21 (DE3) pLysS by IPTG induction, in comparison to the ThiS fusions as control (Figure 5A and B, left panels). Anti-His-tag immunoblot (Figure 5A and B, right panels) of the proteins revealed that MoaD fusions had relatively more proportion in soluble form in comparison to ThiS fusions.

### MoaD fusion protects murine Ribonuclease Inhibitor from degradation

**Figure 6:**
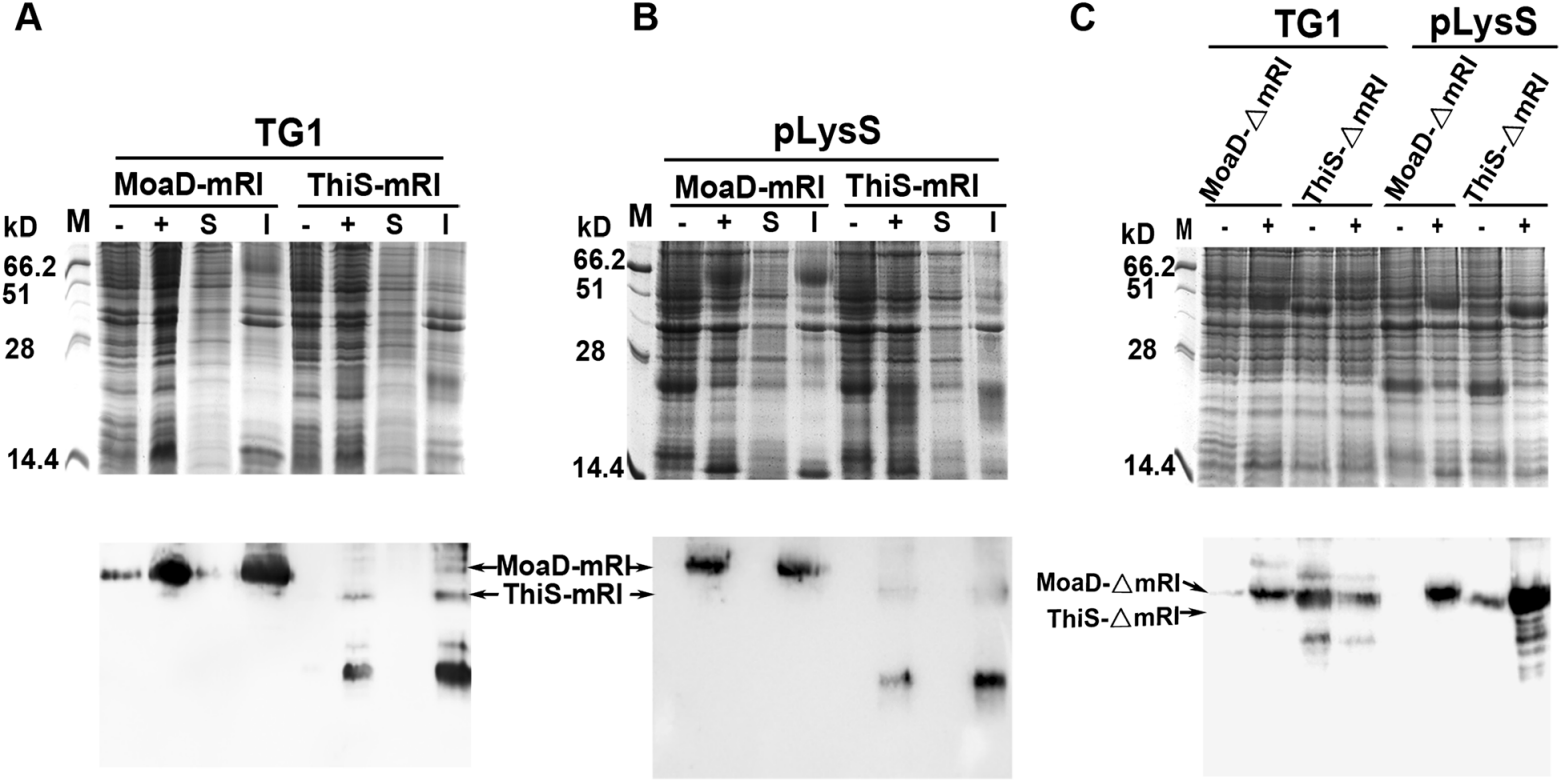
**Expression of mRI and its C-terminal truncated mutant in fusion with MoaD.** mRI with MoaD fusion were expressed in **(A)***E. coli* TG1 or **(B)***E. coli* BL21 (DE3) pLysS. ThiS-fused mRI was used as control. Total cell lysates from uninduced (−) or induced (+) cells with IPTG, and the soluble (S) or insoluble fraction (I) of induced cells were resolved on 10% SDS-PAGE, shown in each upper panel. Western blot probed by anti His-tag antibody was shown in each lower panel. **(C)** C-terminal truncated mRI (△mRI) in fusion with MoaD or ThiS were also expressed in *E. coli* TG1 and BL21 (DE3) pLysS. Expressed products migrating at the expected molecular weight are indicated by arrows.

As fusion of ThiS enhanced the degradation of the unstable protein mRI when expressed in *E. coli*[13], we tried to observe the effect of fusion of MoaD on the stability of mRI. It was shown in SDS-PAGE (Figure 6A and B) that MoaD-fused mRI was expressed at expected molecular weight in inclusion bodies, both in strain TG1 and in the Lon protease deficient *E. coli* strain BL21 (DE3) pLysS. No degraded fragments of MoaD fusion were noticed in Western blots, indicating a complete protection of mRI from degradation by MoaD fusion, in contrast to the enhanced degradation by fusion of ThiS.

It seemed that breakdown occurred more frequently at C-terminus of mRI [13,14]. A mutated mRI, with a merely single mutation which changed Glu340 to a stop coden at C-terminal part in mRI, was fused to MoaD or ThiS. The prematurely stopped and thus slightly shortened products of both fusions were efficiently overexpressed in full length in TG1 and BL21 (DE3) pLysS. Only faintly stained smaller fragments were observed for ThiS fusion (Figure 6C), which was different from that of native mRI in Figure 6A and B. It suggested that target itself determined its degradability *in vivo* when fused to ThiS. The MoaD fusion product remained intact without any degradation. This proved again that MoaD fusion protected the target from degradation.

## Discussion

A variety of fusion tags are used to increase expression yields and change solubility and native folding [15]. However, it is still not clear how fusion tags enhance protein expression. Ub was reported to exert chaperoning effects on fusion proteins, thus increase expression of proteins in *E. coli* and yeast [7,16]. Indeed, Ub has a highly stable structure and is the fastest folding protein known [17]. Thus, it may serve to stabilize and promote proper folding of the fusion target. SUMO, structurally similar to Ub, also promotes folding and structural stability of fusion proteins, and leads to their enhanced expression [18-20].

Prokaryotic MoaD and ThiS share the structure of Ub-like domain with their eukaryotic counterparts Ub and SUMO. Our current results indicate that MoaD confers the enhanced expression of EGFP, either in N-terminal fusion or in C-terminal fusion. In contrast to eukaryotic counterparts Ub and SUMO, MoaD fusion at both N- and C-terminus reduces the soluble protein expression rather than enhances the solubility. This is the same case for ThiS fusion. Instead of promoting its proper folding in refolding experiments, MoaD hinders the native EGFP folding, in a similar way to ThiS. This was not expected because they are stable small proteins with a similar conserved structure as Ub or SUMO. The slowdown of folding, rather than the fast expression of fusion protein, should be a primary factor to drive the expressed fusion protein to inclusion bodies. This was proved by the fact that the most of EGFP with MoaD fusion at C-terminus are aggregated in inclusion bodies as detected by confocal microscopy. Actually, the fast expression of fusion protein should not occur under the leaky expression condition.

Enhancing the production of inclusion bodies is one of the fusion strategies although fewer tags are reported for this purpose [15]. Inclusion bodies protect the products from proteolyses, and usually lead to a higher expression yield. In this study, MoaD did not drive the satisfied overexpression of insulin A and B chains in comparison to the ThiS fusions because of a decreased ability of MoaD in driving the protein aggregation of insulin chains.

Different fusion systems have given variable results of expression [1,3,4], and no single fusion tag is ideal for every protein target. On the expression of unstable mRI, MoaD fusion affords complete protection of the product from intracellular degradation. Ub- and SUMO-fusion of mRI, although leading expression products to inclusion bodies, show a moderate intracellular degradation of products [13]. This difference may be attributed to the rapid aggregation of MoaD fusion product to inclusion bodies that afford protection from proteolytic degradation. On the other hand, ThiS fusion, expected to enhance the rapid aggregation due to the sluggish of folding in a similar way as MoaD fusion, led to the significant degradation of mRI. An active ThiS-directed degradation was anticipated for this unusual observation [13].

## Conclusions

This work shows that MoaD, as a fusion tag, is able to promote expression of some target proteins as expected from its structural similarity to Ub and SUMO. While unexpectedly, MoaD enhances the aggregation of fusion proteins due to a slowdown of refolding, the same as the typical prokaryotic ubl ThiS. Furthermore, MoaD fusion provides an advantage over ThiS in protecting the unstable target from degradation. Hence, in addition to ThiS, MoaD enriches the arsenal of fusion tags in the category of insoluble expression. Expression in inclusion bodies may be required specifically in cases where protein production is toxic to the host cell. It is feasible for the practical use of MoaD as a fusion tag in the expression of some target proteins.

## Methods

### Materials

Biochemicals were purchased from Sigma (St. Louis, MO). Ni-IDA agarose affinity resin was from Vigorous Biotechnology (Beijing, China). Oligonucleotides were from Invitrogen (Shanghai, China). All restriction enzymes and T4 DNA ligase were from TaKaRa (Dalian, China). Pfu DNA Polymerase and LA Taq DNA Polymerase were from Vigorous Biotechnology (Beijing, China).

### *E. coli* strains

*E. coli* TG1 cells were used for cloning, maintenance and propagation of plasmids. TG1 and BL21 (DE3) pLysS cells were used as host for protein expression studies. *E. coli* cells were cultivated in Luria broth under appropriate selective conditions.

### Construction of expression vectors

Standard molecular biology techniques were applied for cloning [21]. All recombinant DNA/constructs were verified by sequencing (Invitrogen, Shanghai, China). All primers used are shown in Table 1.

**Table 1:** Primers used in this study

**Primer**	Primer sequence/featured site*	**PCR product: coding protein**
1: ThiS up	ATAagatctATGCAGATCCTGTTTAACGATC /*Bgl* II	primers 1 + 2: ThiS.
2: ThiS down	ATAgaattcAACCCCCTGCAATAACC /*Eco*R I	
3: ThiS down	ATAggatccCCCTGCAATAACCTGAAAAAG /*Bam*H I	primers 1 + 3: ThiS for fusion to N-terminus of targets
4: MoaD up	ATAagatctATTAAAGTTCTTTTTTTCGCCCAG / *Bgl* II	primers 4 + 5: MoaD for fusion to N-terminus of targets
5: MoaD down	ATAggatccTCCGGTTACCGGCGGG / *Bam*H I	
6: MoaD down	ATActcgagTTAACCTCCGGTTACCGGCGGG /*Xho* I	primers 4 + 6: MoaD for fusion to C-terminus of targets
7: A up	ATAagatctATGGGCATTGTGGAACAGTGCTGCAC /*Bgl* II	primers 7 + 8: insulin chain A
8: A down	ATActcgagTTAGTTGCAATAGTTTTCCAGCTG /*Xho* I	primers 4 + 8: MoaD fusion to A
9: B up	ATAagatctATGTTTGTGAACCAGCATCTGTG /*Bgl* II	primers 9 + 10: insulin chain B
10: B down	ATActcgagTTAGGTTTTCGGGGTATAAAAAAAG /*Xho* I	primers 4 + 10: MoaD fusion to B
11: EGFP up	ATAggatccATGGTGAGCAAGGGCGAGGAGCTG /*Bam*H I	primers 11 + 12: EGFP
		primers 11 + 6: MoaD fusion to C-terminus of EGFP
12: pEGFP-C-3’	TATGGCTGATTATGATCAGT /universal vector primer	primers 11 + 12: EGFP for fusion at C-turminus
13: EGFP down	ATActcgagTCACTTGTACAGCTCGTCCATG /*Xho* I	primers 11 + 13: EGFP for fusion at N-turminus
		primers 1 + 13: ThiS fusion to N-terminal EGFP
		primers 4 + 13: MoaD fusion to N-terminal EGFP
14: RNH up	ATAagatctATGAGTCTTGACATCCAGTGTGAGC /*Bgl* II	primers 14 + 15: mRI
15: RNH down	ATAgtcgacTCAGGAAATGATCCTCAGGGAAGG /*Sal* I	primers 1 + 15: ThiS fusion to ΔmRI
		primers 7 + 15: MoaD fusion to mRI or ΔmRI

*Restriction site in lowercase.

*MoaD* and *ThiS* genes were amplified from genomic DNA of *E. coli* strain TG1. Insulin genes for chain A and B were synthesized as described Yuan *et al.*[13]. Gene fusions were made by restricted fragment ligation. A cDNA of *mRNH* coding mRI (with 456 amino acid residues) [14] and its PCR amplified spontaneous mutant (coding truncated product ΔmRI due to Glu340 was mutated to a stop code) were used for gene fusions.

The expression vectors were based on the pQE30 plasmid (Qiagen, Hilden, Germany) with hexa-His at 5’ fusion, pVI plasmid (Vigilance Biotechnology, Beijing, China) with sept-His at 5’ fusion, or pET28a plasmid (Invitrogen, Carlsbad, CA, USA) with hexa-His at 5’ fusion. All the expression plasmids and their expected products were shown in Table 2.

**Table 2:** Strains and plasmids used in this study

**Strain or plasmid**	**Description**		**Source or reference**
***E. coli *** **strain**	**Host background**		
TG1	K-12 strain		Stratagene
BL21(DE3)pLysS	B strain with Lon protease deficiency, contains pLysS plasmid expressing T7 lysozyme		Novagen
**Plasmid**	**Expressed protein**	MW*	
EGFP/pQE30	EGFP with his-tag at N-terminus, with extra 22 vector sequences.	30578	Previous study [13]
ThiS-EGFP/pQE30	ThiS fused to N-terminus of EGFP with his-tag at N-terminus.	35719	This study
EGFP-ThiS/pQE30	ThiS fused to C-terminus of EGFP with his-tag at N-terminus.	36133	Previous study [13]
MoaD-EGFP/pQE30	MoaD fused to N-terminus of EGFP with his-tag at N-terminus.	37093	This study
EGFP-MoaD/pQE30	MoaD fused to C-terminus of EGFP with his-tag at N-terminus.	37506	This study
ThiS-A/pET28a	ThiS fused Insulin A chain with his-tag at N-terminus.	13438	Previous study [13]
ThiS-B/pET28a	ThiS fused Insulin B with his-tag at N-terminus.	14484	Previous study [13]
MoaD-A/pET28a	MoaD fused Insulin A chain with his-tag at N-terminus.	14812	This study
MoaD-B/pET28a	MoaD fused Insulin B with his-tag at N-terminus.	15858	This study
ThiS-mRNH/pVI	ThiS fused mRI with his-tag at N-terminus.	58661	Previous study [13]
MoaD-mRNH/pVI	MoaD fused mRI with his-tag at N-terminus.	60034	This study
ThiS-ΔRNH/pVI	ThiS fused ΔmRI with his-tag at N-terminus.	45672	This study
MoaD-ΔRNH/pVI	MoaD fused ΔmRI with his-tag at N-terminus.	47045	This study

*Molecular weight of each protein product was calculated based on the predicted protein sequence from corresponding plasmid.

### Expression and purification of recombinant proteins

The culture of *E. coli* was grown overnight and subcultured at 1:100 into Luria broth at 37°C. When the cell growth of the culture reached a mid-log phase, protein expression was induced by adding Isopropyl β-D-1-thiogalactopyranoside (IPTG) to a final concentration of 1 mM, with a further 4 h growth at 37°C, or otherwise indicated. Cells were harvested, resuspended in PBS containing 1% Triton X-100, subjected to cycles of freezing and thawing, and then disrupted using sonication. The soluble protein fraction was separated from insoluble one by centrifugation at 4°C (15 min at 14,000 g). Soluble fraction of His-tagged recombinant proteins were purified by nickel-affinity chromatography under native conditions based on the supplier’s instructions.

### Electrophoresis and Western blot

The samples of whole cells or the purified protein fractions were mixed with Laemmli buffer, either heated in boiling water bath for 5 min or not heated, and analyzed by SDS-PAGE, as described by Laemmli [22], using a 5% stacking gel and a 10% to 15% separating gel run in a Mini-Protean II electrophoresis system (BioRad, Hercules, CA, USA). The gels were stained with Coomassie Blue, or electroblotted onto nitrocellulose or PVDF membranes. His-tagged fusions were detected by immunoblot using anti-His antibody and goat anti-mouse HRP labelled antibody (CoWin Biotech, Beijing, China). Chemiluminescence was recorded using the reagents according to supplier’s protocol (CoWin Biotech, Beijing, China).

### Fluorescence determination of EGFP

The fluorescence of purified soluble EGFPs was measured with excitation wavelength at 488 nm and emission wavelength at 509 nm using EnSpire Multimode Reader (Perkin-Elmer, Waltham, MA, USA). For *E. coli* expressing recombinant EGFP proteins, cultured media containing live whole cells was aliquoted and the fluorescence was measured promptly, the same way as purified proteins. The bacteria concentration of the same sample was also measured as absorbance at 600 nm.

### Confocal microscopy

*E. coli* in LB-medium that expressing recombinant EGFP proteins were dropped onto a slide and sealed with a coverslip. Images were recorded with a confocal laser scanning microscope (Leica TCS SP2, Leica Laser-technik, Heidelberg, Germany) either in phase contrast mode or fluorescence mode (wavelengths at 488 nm for excitation, and 500–560 nm for detection).

### Denaturation and refolding of EGFP

Purified ThiS- or MoaD-tagged EGFP and EGFP without fusion were denatured in PBS containing 8 M urea and 5 mM DTT for 5 min in a boiling water bath. Urea-denatured samples were renatured at room temperature by 10-fold dilution into PBS with 5 mM DTT. Fluorescence recovery was monitored with an interval of 5 s for 50 min. Data were fitted with Sigma Plot (Systat Software, San Jose, CA, USA) and kinetics for fast and slow refolding phases obtained as described [23]. Final refolding was measured at 15 h. The percentage of refolding was calculated on the basis of the final constant amount of fluorescence, corresponding to the amount of fluorescence before denaturation.

### Statistical analysis

The results were derived from three to four independent experiments. The Student’s *t*-test for paired samples was used to calculate the *p* values. The statistical analyses were performed using SPSS 13.0 (IBM SPSS, Armonk, NY, USA), and *p* values less than 0.05 were considered statistically significant.

## Competing interests

The authors declare that they have no conflict of interest.

## Authors’ contributions

SY, XW and JX participated in the design of the study, performed the experiments and data analysis. ZY participated in the experiments. NW conceived of the study, supervised research, participated in the experiments and wrote the paper. All authors read and approved the final manuscript.

## Supplementary Material

Additional file 1: Figure S1 *In vitro* fragmentation of EGFP fused with MoaD at C-terminus. Click here for file
