# Supplementary material for: Ubiquitin-like prokaryotic MoaD as a fusion tag for expression of heterologous proteins in Escherichia coli
Source: BMC Biotechnol. 2014 Jan 21;14:5. doi: 10.1186/1472-6750-14-5 (PMC3906898; doi:10.1186/1472-6750-14-5)
Supplement: Additional file 1: Figure S1 — In vitro fragmentation of EGFP fused with MoaD at C-terminus. [file 1472-6750-14-5-S1.doc]

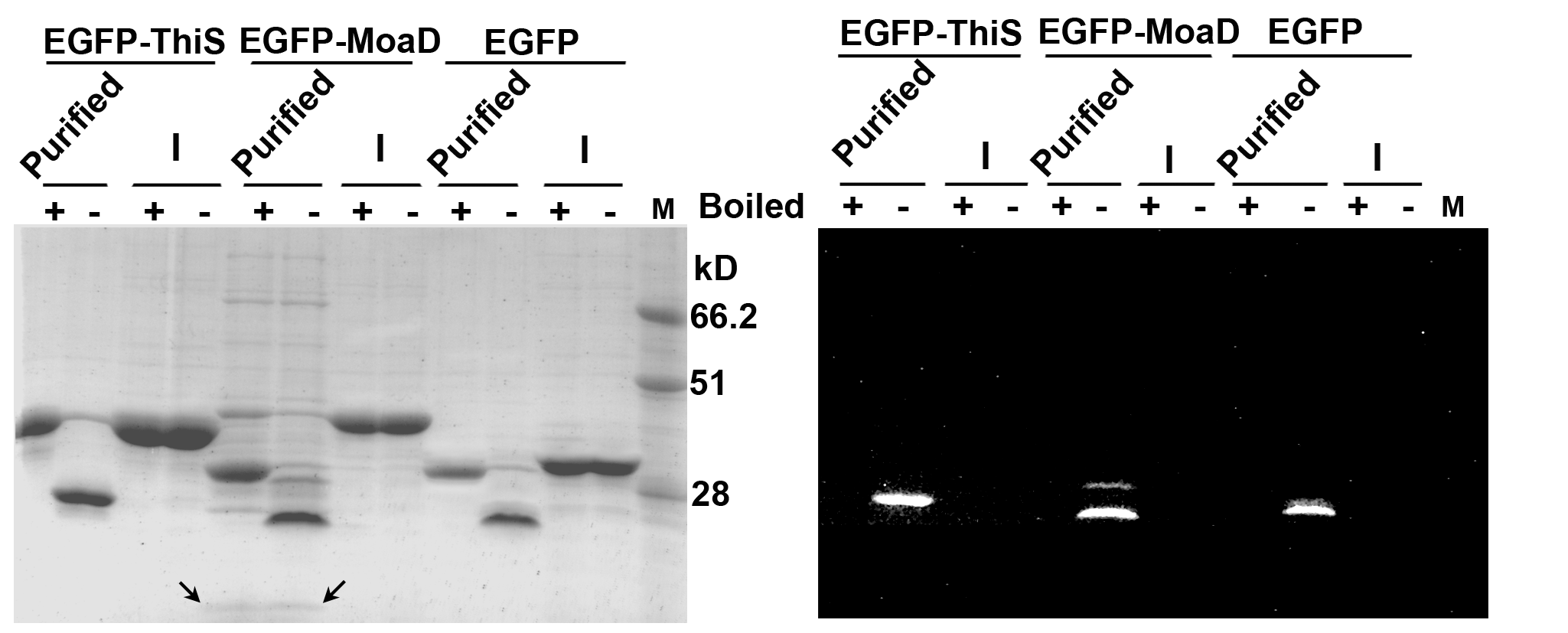


**Figure S1. *In vitro* fragmentation of EGFP fused with MoaD at C-terminus.** Samples of EGFP without or with fusion of ThiS or MoaD at C-terminus, either in active form (Purified) through purification from soluble portion with IMAC, or inactive form (I) solubilized from insoluble portion with urea, were mixed with Laemmli Buffer, loaded onto 12% SDS-PAGE before (-) or after (+) boiling denaturation. Gels were photographed under UV illumination (right panel) prior to Coomassie Blue staining (left panel). EGFP in fusion with C-terminal MoaD lost its integrity only in purified form but not in its inactive form, showed a fluorescent fragment with smaller size than expected which was similar to EGFP along. The C-terminal fragment was retained (possibly the MoaD fragment) in the gel as indicated by arrows, suggesting that this fragmentation occurred *in vitro* after the purification through IMAC. The fragmentation did not occur *in vivo*, as shown in Fig. 2.
